# Pentachlorophenol removal from aqueous solutions by microwave/persulfate and microwave/H_2_O_2_: a comparative kinetic study

**DOI:** 10.1186/2052-336X-12-94

**Published:** 2014-06-11

**Authors:** Ghorban Asgari, AbdolMotaleb Seidmohammadi, Afsane Chavoshani

**Affiliations:** 1 Social Determinants of Health Research Center (SDHRC), Department of Environmental Health Engineering, School of Public Health, Hamadan University of Medical Sciences, Hamadan, Iran; 2 Social Determinants of Health Research Center (SDHRC), Department of Environmental Health Engineering, Gonabad University of Medical Sciences, Gonabad, Iran

**Keywords:** Microwaves, Pentachlorophenol, Hydrogen peroxide, Sodium persulfate

## Abstract

Pentachlorophenol (PCP) is one of the most fungicides and pesticides used in wood protection. Poisoning from PCP may be happened in dermal absorption, and respiration or ingestion. With regard to health and environmental effects of PCP, many methods were studied for its removal. Microwave assisted other methods are environmental friendly, safety, and economical method, therefore, in this study; a modified domestic microwave assisted hydrogen peroxide (MW/H_2_O_2_) and sodium persulfate (MW/SPS) was used for PCP removal from aqueous solutions. PCP removal rate was measured under different factors such as pH, energy intensity, SPS, H_2_O_2_ concentration, Tert- butyl alcohol (TBA) and chemical oxygen demand (COD). The concentration changes of PCP were determined using spectrophotometer and HPLC spectra, respectively. The best removal PCP rate obtained in condition of pH of 11, 0.02 mol L^−1^ of SPS, 0.2 mol L^−1^ of H_2_O_2_ and energy intensity of 600 W. Moreover, COD removals in MW/H_2_O_2_ and MW/SPS process were 83% and 94%, respectively, also TBA test decreased 15% and 3% of PCP removal in MW/SPS and MW/H_2_O_2_ processes respectively. Experimental results indicated that sulfate radical was stronger than hydroxyl radical and examinations order reaction was in first order. In this study, was cleared that MW/SPS process was more effective than MW/H_2_O_2_ process in PCP removal.

## Introduction

PCP, one of the phenolic compounds, is widely used in Wood protective industry [1]. Exposure of this compound makes diseases such as aplastic anemia, leukemia, peripheral neuropathy and other problems related to nerve damage (neurotoxicity). This pollutant is a significant contaminant of soil, surface, and groundwater especially around wood preserving facilities [2-6]. Researchers using a mathematical model calculated that 96.5% of PCP is in soil, 2.5% in water, 1% in air, and less than 1% in suspended sediments and organisms in aquatic environments [6,7]. Therefore, PCP removal from aqueous solutions is essential. According to previous studies is cleared that conventional treatment methods are ineffective for PCP and other refractory compounds removal, because these methods can only transfer the contaminants from one phase to another producing many environmental problems [8]. Recently researchers have found that microwave (MW) heating in combination with hydrogen peroxide (H_2_O_2_) and sodium persulfate (Na_2_S_2_O_8_ or SPS) [9] can mineralize organic compounds successfully and completely [6,10,11]. The key effects of these processes is the replacement of hazardous solvents with environmentally benevolent ones [8]. Basic of MW process is the ability of molecules or substances to absorb and transmit MW irradiation [12]. MW irradiation is electromagnetic irradiation in the frequency range of 0.3 to 300 GHz, but laboratory microwave reactors operate at frequency of 2.45 GHz [6,13,14]. By breaking oxygen–oxygen bonds of H_2_O_2_ and S_2_O_8_^2−^, MW commonly are able to dissociate H_2_O_2_ and S_2_O_8_^2−^ into OH^0^ and SO_4_^0^ radicals and other radicals which are very powerful oxidizing species [15].

Similar to hydroxyl radicals, sulphate radicals react with organics by electron transfer, hydrogen abstraction, or addition mechanisms [16,17]. According to results obtained of previous studies, SPS and H_2_O_2_ could be a good option for the MW oxidation technique. In this study, due to an environmental-friendly in addition to highly efficient method and low existence of specific work in this condition, analysis of the PCP removal by MW/H_2_O_2_ and MW/SPS under various kinds of parameters was performed and in the end, the effectiveness of MW/SPS and MW/H_2_O_2_ processes in the PCP removal was compared.

## Materials and methods

### Materials

Sodium salt PCP, which is the sodium salt of PCP (C_6_C_l5_ONa) with 98% purity was used without further purification. The characteristics of the PCP included of boiling point: 309-310C^0^, mass molar: 288.32 g mol^−1^. PCP solution was prepared by dissolving PCP in NaOH solution to accelerate its dissolution [6,18]. Hydrogen peroxide (30% w/w) and the sodium persulfate from Merck, 98% mass molar: 238.1 g mol^−1^ were used as oxidants.

### Experimental methods and measurements

**Figure 1:**
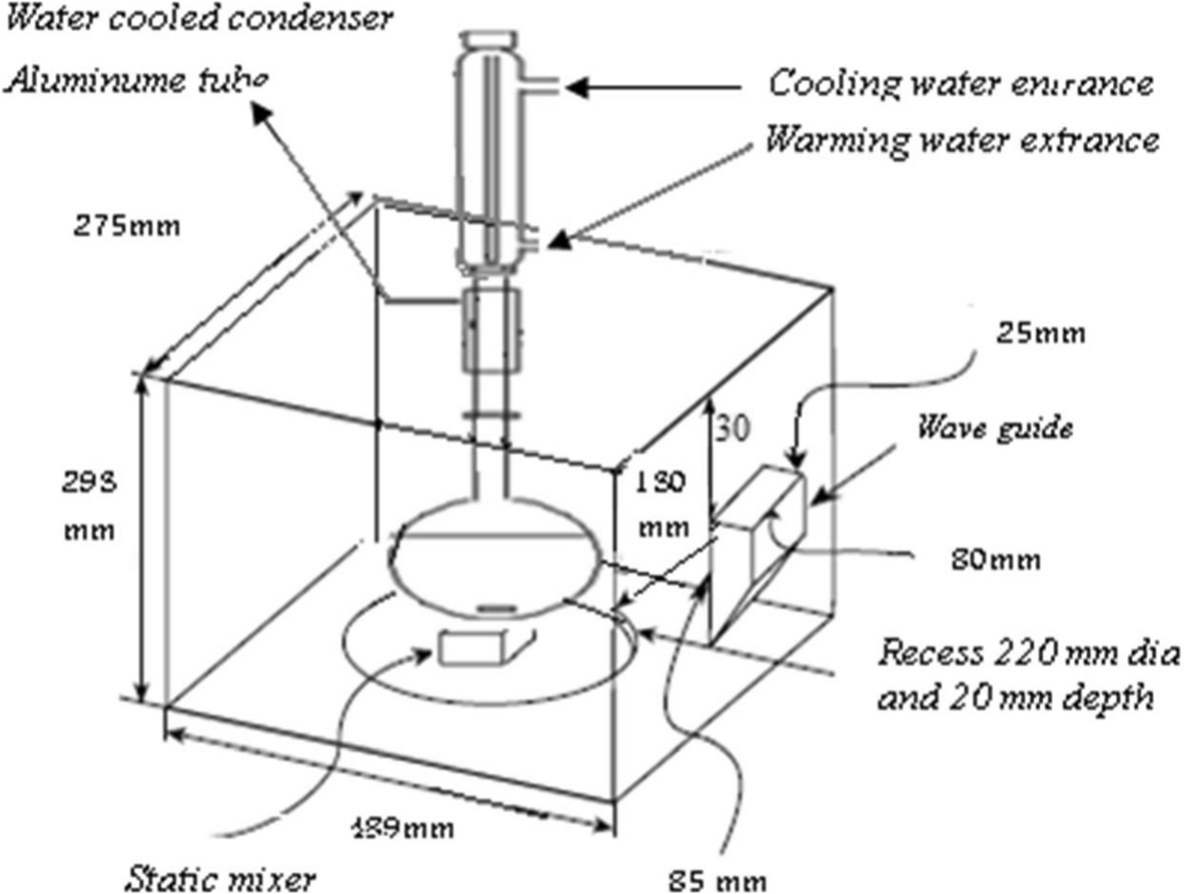
Schematic diagram of modified microwave system.

Under atmospheric pressure, all of the experiments were performed and triplicated in modified domestic microwave oven (2450 MHz, M2330 DN, SAMSUNG Co, and output power of 100 to 850 W) (Figure 1). Detail modifications of MW were presented as follows: drilled a hole in the upper oven wall and then attached an aluminum tube of the same diameter to the hole then equipped with cooling system and a glass reactor with 500 mL volume. Then Samples were taken at suitable time intervals (10 min) from the reaction reactor with a 10 mL syringe and pipetted in to glass vials [6,11]. Besides, a Thermometer GENWAY Medal 2003 was utilized to detect variation of solution temperature during degradation process. The leakage of MW oven is measured at 20 cm distance from the aperture.

In this study different factors effects such as pH (3, 7, 11), energy intensity (180,450,600 W with optimal temperatures 80, 100, and 105C^0^ respectively), SPS and H_2_O_2_ dose (0.01, 0.02, 0.03, 0.04, 0.05 mol L^−1^), PCP concentration (100, 200, 300, 400, 500, 750, 1000 mg L^−1^), effect of Tert-butyl alcohol (TBA ) with 0.04 mol L^−1^ concentration, and COD (344 mg L^−1^) were determined. Changes of PCP concentration were detected using spectrophotometer according to (APHA [19]), and HPLC. HPLC (Part Number.WATO54275 with dimension of 4.6 mm × 250 mm and column of symmetry C18-50 μm) method was performed with an acetonitrile/water 60:40 (v/v) as mobile phase at a flow rate of 1 mL min^−1^ and detection wavelengths of UV was 254 nm [6,20]. COD was detected using potassium dichromate solution as oxidizer in a strong acid medium, then by titration step using ferrous ammonium sulfate as the reducing agent and Ferroin as the indicator [6,9].

## Results and discussion

### Effect of pH on PCP removal

**Figure 2:**
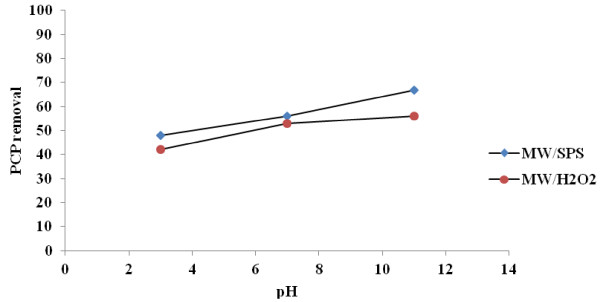
**Effect of pH on PCP removal under MW/SPS and MW/H**_**2**_**O**_**2 **_**systems (PCP = 100 mg L**^**−1**^**, E = 600W, SPS and H**_**2**_**O**_**2 **_**dose = 0.02 mol L**^**−1**^**, reaction time = 30 min).**

In this study under MW/SPS and pHs of 3,7and 11, PCP removal rate was 48, 56, 67% respectively, but under MW/H_2_O_2_, its amount was 42, 53 and 56% respectively (Figure 2). It seems that strong power of MW in ionization of SPS and H_2_O_2_ leads to a negligible difference between all pHs effect (Figure 2), therefore more research for pH effect is necessary. However, results shown that alkaline pH could more accelerate PCP degradation in MW/SPS and MW/H_2_O_2_ systems. These phenomena under MW/SPS and MW/H_2_O_2_ were attributed to the ability of H_2_O_2_ and SPS to absorb and transmit microwave irradiation in alkaline pH more a little than other pHs. Subsequently more radicals are produced in this condition [21-25].

In general and according to experimental conditions following reactions can be performed:

Under MW/SPS:

(1) $$\mathrm{All}\;\mathrm{pHs}:{{\mathrm{SO}}_{4}}^{0-}+H_{2}O\to {{\mathrm{SO}}_{4}}^{-2}+{\mathrm{OH}}^{0}+H^{+}$$

(2) $$\mathrm{Alkaline}\;\mathrm{pH}:{{\mathrm{SO}}_{4}}^{0-}+{\mathrm{OH}}^{-}\to {{\mathrm{SO}}_{4}}^{-2}+{\mathrm{OH}}^{0}$$

Under MW/H_2_O_2_:

(3) $$H_{2}O_{2}+\mathrm{MW}\to 2{\mathrm{OH}}^{0}$$

(4) $${\mathrm{OH}}^{0}+H_{2}O_{2}\to H_{2}O+{{\mathrm{HO}}_{2}}^{0}$$

(5) $$2{\mathrm{OH}}^{0}\to H_{2}O_{2}$$

(6) $$2{\mathrm{OH}}^{0}\to H_{2}O_{2}+O_{2}$$

(7) $${\mathrm{OH}}^{0}↔{O_{2}}^{-}+H^{+}$$

(8) $$H_{2}O+{{\mathrm{HO}}_{2}}^{0}+{O_{2}}^{-}\to H_{2}O_{2}+O_{2}+\mathrm{OH}$$

Under MW/SPS, the rate constants for Eqs. (1) and (2) are < 2 × 10^−3^ and (6.5 ± 1) × 10^7^ M^−1^S^−1^ respectively. It is cleared that the reaction rate constant of Eq. (2) is more than Eq. (1). According these equations in all pHs and alkaline pH, both SO_4_^0−^ and OH^0^ are possibility responsible for degradation of organic contaminants, but previous studies have shown that in pHs of 3–10, amount of hydroxyl radical is more than sulfate radical and in pH > 10.5 amount of sulfate radical is more than hydroxyl [26,27]. According these results, the difference between our work and previous studies could partly attribute to pH = 11. Results of other studies confirm that organic removal efficiency is more in alkaline pH [15]. In similar to, under MW/H_2_O_2_ in alkaline pH, amount of OH^0^ and other radicals participating in PCP removal is more than other pHs (Eqs (3) to (8)) [6,11,28].

### Effect of SPS and H_2_O_2_ concentrations on PCP removal

**Figure 3:**
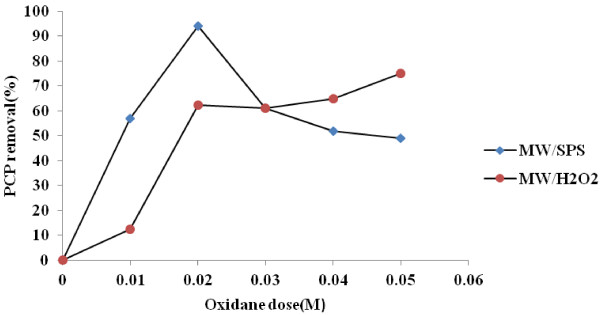
**Effect of oxidant concentration on PCP removal under MW/SPS and MW/H**_**2**_**O**_**2 **_**systems (pH = 11, PCP = 100 mg L**^**−1**^**, E = 600 W, reaction time = 30 min).**

From Figure 3 is observed that under MW/SPS with increasing SPS concentration from 0.01 to 0.0 2 mol L^−1^, PCP removal efficiency was increased (56 to 94%). But with increasing the initial SPS concentration from 0.02 to 0.05 mol L^−1^ PCP removal rate was decreased from 94 to 49% respectively. Furthermore under MW/H_2_O_2,_ PCP removal efficiency for 0.01 to 0.05 mol L^−1^ of H_2_O_2_ was 12.5% to 75% respectively. PCP removal (87%) was stabled in doses of 0.2 and 0.3 mol L^−1^ of H_2_O_2_ (data not show). Therefore, optimal doses of SPS and H_2_O_2_ were 0.02 and 0.2 mol L^−1^ respectively. Shih et al. reported that, in extremely high initial concentration, SO_4_^0−^ reacted with persulfate according to the following equation [29].

(9) $${{\mathrm{SO}}_{4}}^{0-}+S_{2}{O_{8}}^{2-}\to {{\mathrm{SO}}_{4}}^{2-}+S_{2}{O_{8}}^{0-}$$

So that an over-dose of persulfate transformed the SO_4_^0−^ to S_2_O_8_^0−^ reducing the oxidizing power for PCP removal [30,31]. Also with respect to Eq (10), under high H_2_O_2_ concentration in MW/H_2_O_2_ system, quenching of OH° radicals is happened to produce HO_2_° radicals [6,11,15].

(10) $$H_{2}O_{2}+OH^{0}\to H_{2}O+HO_{2}^{0}$$

Therefore, existences of a scavenger of OH° radicals, such as H_2_O_2,_ have a decreasing effect in the organic compounds removal efficiency [6,17].

### Effect of different energy intensity on PCP removal

**Figure 4:**
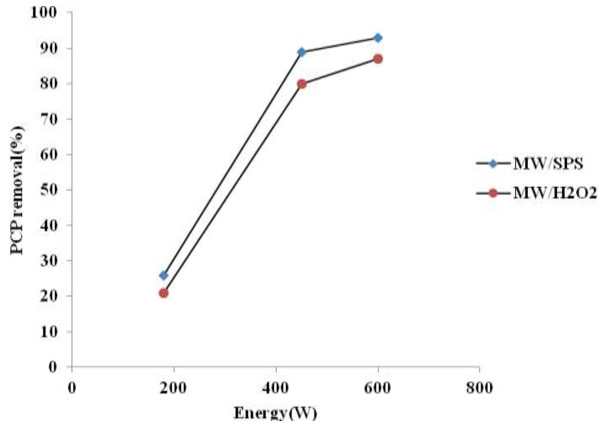
**Effect of energy intensity on PCP removal under MW/SPS and MW/H**_**2**_**O**_**2 **_**systems (pH = 11, PCP = 100 mg L**^**−1**^**, SPS and H**_**2**_**O**_**2 **_**dose = 0.02 and 0.2 mol L**^**−1**^**, reaction time = 30 min).**

The test results shown in Figure 4 indicated that PCP removal efficiency gradually increased by increasing the microwave power from 180 to 600 W. Amount of PCP removal in MW/SPS with energy intensity of 180, 450, and 600 W was 26, 89 and 93%, respectively. In addition, under MW/H_2_O_2_ system amount of PCP removal was 20, 80 and 87%, respectively. PCP removal efficiency did not change for higher power (>600 W). Subsequently, the microwave irradiation of 600 W was chosen for further experiments. According to other studies, removal efficiency can only increase to a limited extent [32] and degradation of organic materials is not always increased with increasing microwave power [33,34]. In this study, amount of Energy consumption in optimal condition (energy power of 600 W and reaction time of 30 min) for both of systems was 0.3 KWh , also other researchers confirm that energy consumption in MW process is very low and economy [35,36].

### Effect of radical scavenger on PCP removal

**Figure 5:**
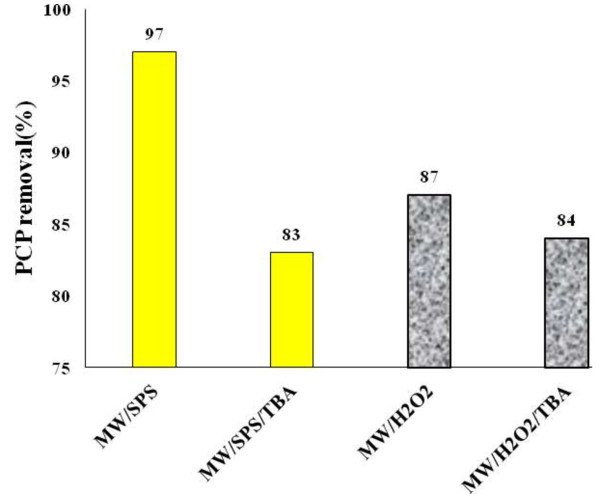
**Effect of TBA on PCP removal under MW/SPS and MW/H**_**2**_**O**_**2 **_**systems (pH = 11, PCP = 100mg L**^**−1**^**, SPS and H**_**2**_**O**_**2 **_**dose = 0.02, 0.2 mol L**^**−1**^**, TBA = 0.04 mol L**^**−1**^**, E = 600 W, reaction time = 30 min).**

In this study, 0.04 mol L^−1^ TBA (OH^0^ scavenger) added to MW/SPS and MW/H_2_O_2_. The results shown that the degradation rate of PCP was decreased 15% and 3% under MW/SPS/TBA and MW/H_2_O_2_/TBA respectively (Figure 5). According to Eqs. (11) to (16) [37] is cleared that both SO4^0−^ and OH^0^ can degrade PCP, but with respect to TBA test, SO_4_^0−^ in MW/SPS play the dominant role and OH^0^ had only a negligible role (15%).

(11) $$-O_{3}S-O-O-S-{O^{-}}_{3}\to 2-O_{3}S-O^{0}$$

(12) $$-O_{3}S-O^{0}+e^{-}\to {{\mathrm{SO}}_{2}}^{-4}$$

(13) $$-O_{3}S-O^{0}+\mathrm{PCP}\to \mathrm{Products}$$

(14) $$-O_{3}S-O^{0}+H_{2}O\to {\mathrm{HSO}}^{-4}+{\mathrm{OH}}^{0}$$

(15) $$-O_{3}S-O^{0}+{\mathrm{HO}}^{-}\to {{\mathrm{SO}}_{2}}^{-4}+{\mathrm{OH}}^{0}$$

(16) $${\mathrm{OH}}^{0}+\mathrm{PCP}\to \mathrm{Products}$$

MW/H_2_O_2_/TBA results show that OH^0^ is activation initiator and isn’t dominant radical (its role was only 3%). Based on following equations, MW is able to dissociate H_2_O_2_ to many radicals as well as OH^0^[6,28,38]. Also according to Hong et al. results in MW/H_2_O_2_ system, O_2_ is dominant radical [9].

(17) $$H_{2}O_{2}+\mathrm{MW}\to 2OH^{0}$$

(18) $$OH^{0}+H_{2}O_{2}\to H_{2}O+H{O_{2}}^{0}$$

(19) $$2OH^{0}\to H_{2}O_{2}\;$$

(20) $$2OH^{0}\to H_{2}O_{2}+O_{2}\;$$

(21) $$OH^{0}↔{O_{2}}^{-}+H^{+}$$

(22) $$H_{2}O+H{O_{2}}^{0}+{O_{2}}^{-}\to H_{2}O_{2}+O_{2}+OH^{-}\;$$

### Reaction kinetics

**Figure 6:**
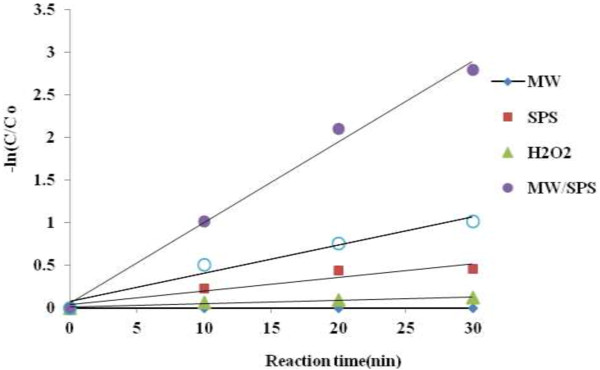
**Reaction kinetics of PCP removal under different processes (pH = 11, PCP = 100 mg L**^**−1**^**, SPS, H**_**2**_**O**_**2 **_**dose = 0.02, 0.2 mol L**^**−1**^**, E = 600 W, reaction time = 30 min).**

Obtained Results from reaction kinetics under MW/SPS and MW/H_2_O_2_ demonstrated that the PCP removal follows first-order kinetics law (Figure 6). In this study, K SPS and K H_2_O_2_ only was 0.014 min^−1^ and 0.004 min^−1^ respectively, but K MW/SPS and K MW/H_2_O_2_ was 0.095 min^−1^ and 0.055 min^−1^ respectively. So that, K SPS was 3.5 times more than K H_2_O_2_, and K MW/SPS was 1.72 times more than K MW/H_2_O_2_. Because energy of oxygen-oxygen bond in persulfate is less than H_2_O_2_[15], SPS activation and subsequently PCP removal occur more rapidly in MW/SPS system than MW/H_2_O_2_. Also synergetic factor of MW in MW/SPS and MW/H_2_O_2_ was 6.6 and 13.75 respectively. This factor shows that MW process have higher synergetic effect on H_2_O_2_ decomposition than SPS [39].

### Mineralization of PCP in MW/SPS and MW/H_2_O_2_ processes and identification of oxidation intermediates

**Figure 7:**
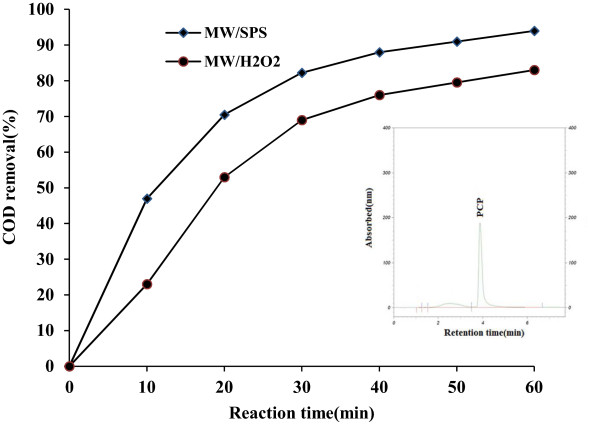
**Mineralization of PCP in MW/SPS and MW/H**_**2**_**O**_**2 **_**processes and identification of oxidation intermediates (pH = 11, COD = 344 mg/L, SPS and H**_**2**_**O**_**2 **_**dose = 0.02 and 0.2 mol L**^**−1**^**, E = 600 W).**

Results abstained from COD removal showed that MW/SPS and MW/H_2_O_2_ were able to remove COD in amount of 94 and 83% respectively (Figure 7). Intermediates detected via HPLC were CO_2_ and HCL (Eq. 23). In this study, the HPLC spectra and COD results showed a similar trend in mineralization and the lack of toxic intermediates and by products [28,39].

(23) $$C_{6}{\mathrm{HCL}}_{5}O+\mathrm{MW}\to {\mathrm{CO}}_{2}+5\mathrm{HCl}$$

## Conclusion

MW/SPS and MW/H_2_O_2_ processes could efficiently degrade refractory compounds at strong alkaline, via radical production. MW/SPS in PCP removal was more effective than MW/H_2_O_2_, because SPS is dissociated and activated more easily than hydrogen peroxide. Addition of SPS and H_2_O_2_ doses during MW process enhances the rate of PCP degradation, except when the radical scavenging effects of SPS and H_2_O_2_. Results obtained from radical scavenger test showed that OH° had only an initiator role, and had not a dominant role and order reaction in both of systems was in first order. Also, the microwave degradation is able to mineralize refractory compounds without any toxic byproduct. The microwave degradation has many advantages such as convenience, safety, economy and high efficiency. Accordingly these methods, especially WM/SPS, have a better prospect in future for removal of other chlorinated organic compounds such as Aldrin, Dieldrin and Lindane, in alkaline pH.

## Abbreviations

PCP: Pentachlorophenol; AOPs: Advanced oxidation processes; TBA: Tert- butyl alcohol; COD: Chemical oxygen demand; SPS: Sodium Persulfate; H_2_O_2_: Hydrogen Peroxide; MW: Microwave; K: Reaction constant.

## Competing interests

The authors declare that they have no competing interests.

## Authors’ contributions

All authors studied and approved the final manuscript.
